# Supplementary material for: aYAP modRNA reduces cardiac inflammation and hypertrophy in a murine ischemia-reperfusion model
Source: Life Sci Alliance. 2019 Dec 16;3(1):e201900424. doi: 10.26508/lsa.201900424 (PMC6918510; doi:10.26508/lsa.201900424)
Supplement: Supplementary file 1 [file LSA-2019-00424_Supplemental_Data_1.doc]

SUPPLEMENTAL MATERIAL

Supplemental Methods

Cardiac ischemia reperfusion (IR) surgery and modRNA intramyocardial injection

After anesthetized with isoflurane, mice were fixed on a sterile surgery board. The surgery board was kept around 37°C throughout the whole procedure. The chest was shaved and cleaned with alcohol. A suture was placed around the front upper incisors and pulled taut so that the neck is slightly extended. The tongue was retracted and held with forceps, and a 20-G catheter is inserted into the trachea. The catheter was then attached to the mouse ventilator via a Y-shaped connector. Ventilation was performed with a tidal volume of 200 µl and a respiratory rate of 133/min. 100% oxygen was provided to the inflow of the ventilator. Prior to the incision, lidocaine is introduced under the skin. The chest cavity was opened by an incision of the left second intercostal space. Chest retractor was applied to facilitate the view. The pericardial sac was opened and dissected apart. The left anterior descending artery was ligated using 7-0 silk suture and a thin metal tube was placed within the knot. To reduce the LAD surgery variation, we used red fluorescence beads to indicate the coronary blood flow. Area with no fluorescence beads was defined as area at risk. 3-5 minutes after LAD ligation, sterile fluorescent microbeads (Catalog Number: F8834, Life Technology) were injected into the left ventricular cavity to determine the area of perfused myocardium. Then 75 µl Yap modRNA (0.67µg/µl in 0.9% NaCl) or same volume of vehicle solution (0.9% NaCl) was injected into 3 sites of the infarction border zone with a ultra-fine needle insulin syringe (BD, Cat: 324909). 50 minutes after LAD ligation, the knot was released to reperfuse the myocardium. The chest cavity, muscles, and skin were closed layer by layer. During the procedure, lungs were slightly overinflated to assist in removal of air in the pleural cavity. In the sham control group, LAD ligation and modRNA/Vehicle injection were not processed, and the other procedures were the same as the IR groups.

Histology and immunostaining

Hearts were fixed in 2% PFA, dehydrated with ethanol, and embedded in paraffin. 7-μm sections were used for H&E staining, Sirius Red/Fast Green staining and immunostaining. Images were quantified using Image J software.

For chromogenic immunohistochemical staining on paraffin sections, 7-μm sections were deparaffinized, rehydrated, peroxidase blocked, and antigen exposed by boiling in Target Retrieval Solution (Dako). Signals were detected using the DAB Substrate Kit or ImmPRESS®-AP Anti-Rabbit IgG (alkaline phosphatase) Polymer Detection Kit (Vector Laboratories). Imaging was performed on Keyence microscope. Both low (2x) and high magnification(40x) images were taken to quantify the neutrophil or macrophage infiltration. For immunofluorescence staining on paraffin sections, 7-μm sections were deparaffinized, rehydrated, and antigen exposed by boiling in Target Retrieval Solution (Dako). Immunofluorescence staining was visualized on a Fluoview 1000 confocal microscopy system.

Flow cytometry analysis

The heart apex was used for dissociation with a published protocol^1^ Non-CMs suspensions were filtered through 40-μm cell strainers. The cells were washed with PBS containing 2% FBS. Antibodies used for flow cytometry analysis included the following: PE-conjugated CD45 (30-F11, eBioscience), Alexa APC-conjugated CD11b (M1/70,Biolegend), PE/Cy7-conjugated Ly6G(1A8,Biolegend), Percp-conjugated F4/80 (BM8,Biolegend). Unstained cells were used as negative control to establish the flow cytometer voltage setting, and single-color staining controls were used for adjustment of the compensation. Flow cytometer was performed on CANTO II (BD). The flow cytometric data were analyzed with FlowJo software (TreeStar) and DIVA software (BD). Investigator doing staining and flow cytometry analysis was blind to the modRNA treatment.

**Adenovirus** **And Neonatal rat ventricular myocytes (NRVMs)**

LacZ and aYAP were described previously (von Gise et al., 2012). Adenovirus expressing HA tagged CD14 was purchased from Applied Biological Materials (abm) Inc.

Neonatal rat ventricular myocytes (NRVMs) were isolated from 2-day-old Wistar rats (Charles River) using the Neomyts cardiomyocyte dissociation kit (Cellutron). Isolated cardiomyocytes were initially cultured for 24 hours in the presence of 10% fetal bovine serum (NS medium, Cellutron). Cardiomyocytes were then cultured in NW (Cellutron) medium containing 5% horse serum and 20 μM cytosine β-D-Arabinofuranoside (AraC;Sigma) for 48 hours. After washing with PBS, medium was changed into serum free NW medium, and NRVMs were infected with adenovirus at 20 MOI. 12 hours later, cells were washed with PBS and then cultured in NW medium containing 1µg/ml LPS for 24 hours before experiments. LacZ infected cells with no LPS treatment served as negative control.

Adult cardiomyocytes isolation and treatment

Adult Ventricular myocytes were isolated from 6–8 week old C57/BL6J mice (Jackson Lab) following a previously described method.^2^ After isolation, adult cardiomyocytes were maintained in cardiomyocytes medium (ScienCell) at 37°C for 2 hours, allowing cell attachment to laminin-coated 6 well plate. After 2 hours, the cells were replaced with cardiomyocytes medium and treated with adenovirus overnight. On the second day, to mimic the oxygen stress of ischemia/reperfusion, adult cardiomyocytes were treated with 50µM H_2_O_2_ for 6 hours. To induce innate immune response, adult cardiomyocytes were treated with 1µg/ml LPS for 24 hours. At the end of the treatments, adult cardiomyocytes were either fixed with 4% PFA or collected for RNA isolation.

Adult cardiomyocytes necrosis detection

Adult cardiomyocytes cultured on 48-well plates were used for necrosis study. Briefly, propidium iodide (PI) 1µg/ml was directly added into the culture medium. Cells were incubated for 10 minutes at 37°C, and were directly used for imaging with Keyence microscope.

Myocardium cell death analysis

TUNEL staining (Click-iT^TM^ Plus TUNEL assay, Invitrogen) was performed on paraffin-embedded sections at 2 days after IR. Hearts from animals not receiving EdU were used for this staining. To quantify TUNEL staining sections, sections were photographed at low magnification using Keyence microscope. The total TUNEL positive area was quantified using Image J software.

CM necrosis was analyzed based on Mf20 antibody (DSHB) permeability. 1 day after IR, animals were intraperitoneally injected with 100µl Mf20 antibody supernatant. 18-24 hours later hearts were collected and fixed with 2% PFA. Paraffin sections were stained with Alexa 488 conjugated Donkey anti mouse secondary antibody to visualized Mf20 positive cardiomyocytes.
