# Supplementary material for: aYAP modRNA reduces cardiac inflammation and hypertrophy in a murine ischemia-reperfusion model
Source: Life Sci Alliance. 2019 Dec 16;3(1):e201900424. doi: 10.26508/lsa.201900424 (PMC6918510; doi:10.26508/lsa.201900424)
Supplement: Supplementary file 2 [file LSA-2019-00424_TableS1.doc]

Supplemental Table 1 Antibodies used for immunostaining and western blot

| Antigen | Company (catalog #) | Origin | Working dilution |
| --- | --- | --- | --- |
| Primary antibodies | |  |  |
| Mac-3 | BD (553322) | Rat | 1:50 for IHC |
| Ly-6G | BD (551459) | Rat | 1:50 for IHC |
| Cardiac troponin I (TNNI3) | Abcam (ab56357) | Goat | 1:200 for IF |
| WGA-647 | Life Technology (W32466) | NA | 1:250 for IF |
| Flag | Sigma (F1804) | Mouse | 1:100 for IF |
| Tlr4 | Santa Cruz Biotechnology | Mouse | 1:500 for western blot |
| GAPDH | Santa Cruz Biotechnology | Rabbit | 1:1000 for western blot |
|  |  |  |  |
