# Supplementary material for: aYAP modRNA reduces cardiac inflammation and hypertrophy in a murine ischemia-reperfusion model
Source: Life Sci Alliance. 2019 Dec 16;3(1):e201900424. doi: 10.26508/lsa.201900424 (PMC6918510; doi:10.26508/lsa.201900424)
Supplement: Supplementary file 3 [file LSA-2019-00424_TableS2.doc]

Supplemental Table 2 Primers used for qRT-PCR

| Gene name | Species | Forward | Reverse |
| --- | --- | --- | --- |
| Tlr1 | Mouse | GGTTGTCTTGACGGAACACG | TTCTTCAGAGCATTGCCACA |
| Tlr2 | Mouse | GAGGTGCGGACTGTTTCCTT | AGATTTGACGCTTTGTCTGAGG |
| Tlr3 | Mouse | AATCCTTGCGTTGCGAAGTG | GGTTCAGTTGGGCGTTGTTC |
| Tlr4 | Mouse | CCTGACACCAGGAAGCTTGA | TCCAGCCACTGAAGTTCTGA |
| Tlr5 | Mouse | TCACTGCATACCTGGTTCCC | TTGACATGCCATGATCCTGCT |
| Tlr6 | Mouse | GGTACCGTCAGTGCTGGAAA | TATTAAGGCCAGGGCGCAAA |
| Nppa | Mouse | CTCTGGATTGGTCTCCCAGC | GTCATTCTGTCACTCAAACTCTGG |
| Myh6 | Mouse | CTCTGGATTGGTCTCCCAGC | GTCATTCTGTCACTCAAACTCTGG |
| Gapdh | Mouse | CAGGTTGTCTCCTGCGACTT | GGCCTCTCTTGCTCAGTGTC |
| Cd14 | Mouse | CTCTGTCCTTAAAGCGGCTTAC | GTTGCGGAGGTTCAAGATGTT |
| Yap | Mouse/Human | ATTGTTCTCAATTCCTGAGAC | CAGGTTGTCTCCTGCGACTT |
| Il-12a | Mouse | GATGACATGGTGAAGACGGC | AGGCACAGGGTCATCATCAA |
| Ccl2 | Mouse | GTTGGCTCAGCCAGATGCA | AGCCTACTCATTGGGATCATCTTG |
| Il-6 | Mouse | CACTTCACAAGTCGGAGGCT | CTGCAAGTGCATCATCGTTGT |
| Il-10 | Mouse | GGTGAGAAGCTGAAGACCCTC | GCCTTGTAGACACCTTGGTCTT |
